# The effect of paternal cues in prenatal care settings on men’s involvement intentions

**DOI:** 10.1371/journal.pone.0216454

**Published:** 2019-05-09

**Authors:** Analia F. Albuja, Diana T. Sanchez, Shawna J. Lee, Joyce Y. Lee, Stacy Yadava

**Affiliations:** 1 Department of Psychology, Rutgers University, New Brunswick, New Jersey, United States of America; 2 School of Social Work, University of Michigan, Ann Arbor, Michigan, United States of America; 3 Department of Obstetrics, Gynecology and Reproductive Sciences, Robert Wood Johnson Medical School, New Brunswick, New Jersey, United States of America; Sefako Makgatho Health Sciences University, SOUTH AFRICA

## Abstract

A father’s involvement in prenatal care engenders health benefits for both mothers and children. While this information can help practitioners improve family health, low paternal involvement in prenatal care remains a challenge. The present study tested a simple, easily scalable intervention to promote father involvement by increasing men’s feelings of comfort and expectations of involvement in prenatal settings through three randomized control trials. Borrowing from social psychological theory on identity safety, the three studies tested whether the inclusion of environmental cues that represent men and fatherhood in prenatal care offices influenced men’s beliefs and behavioral intentions during the perinatal period. Men in studies 1 and 3 viewed online videos of purported prenatal care offices, while men in study 2 visited the office in person. Those who viewed or were immersed in a father-friendly prenatal care office believed that doctors had higher expectations of father involvement compared to treatment-as-usual. This perception predicted greater parenting confidence, comfort, and behavioral intentions to learn about the pregnancy and engage in healthy habits, such as avoiding smoking and alcohol during their partner’s pregnancy. Study 3 replicated these studies with an online sample of expectant fathers. The results suggest that shifting environment office cues can signal fathering norms to men in prenatal settings, with healthier downstream behavior intentions.

## Introduction

Prenatal father involvement, wherein expectant fathers engage in behaviors that support the likelihood of positive pregnancy outcomes, benefits both mothers and infants [1]. For example, father involvement during pregnancy is associated with increased receipt of prenatal care, reduced maternal alcohol and tobacco use, and a lower likelihood of low birth weight infants [2,3]. Moreover, involvement during the prenatal period is a strong predictor of involvement later in the child’s life, with continued positive outcomes [4,5]. Despite these benefits, father involvement during pregnancy is often low with few known interventions to engage expectant fathers [6]. Given that involved fathers engender a host of positive outcomes, the present studies tested a simple, scalable intervention to promote father involvement by improving men’s comfort and expectations in prenatal settings.

There are many barriers to fathers’ involvement during pregnancy, including socioeconomic, interpersonal, and motivational factors [6]. For example, men are less likely to be involved during pregnancy if they are unemployed [7] and have lower income [8]. Other work illustrates the importance of the parents’ relationship in predicting fathers’ prenatal involvement; men are less likely to be involved during the pregnancy if they have more conflict with the mother [9] or if the couple is not currently romantically involved or cohabitating [7]. Lastly, other research finds men with less egalitarian gender attitudes are less involved [10].

However, one understudied factor is the climate surrounding expectations of fathers’ roles, including men’s perceptions of the importance of father involvement. Fathers may demonstrate low prenatal involvement because salient social norms do not hold men to high involvement expectations. Based on the social roles ascribed to men and women by society, men are held to lower expectations as caregivers compared to women [11]. Whereas women are strongly prescribed to be warm, kind, and interested in children, men are prescribed to be interested in their careers [12,13]. These prescriptions are reinforced through implicit associations between women and parenting, and men and careers [14]. Fathers are seen as the secondary parent and are expected to perform fewer caregiving tasks [15,16]. In fact, common conceptualizations of fatherhood did not include a nurturing role for fathers until the mid-1970s [17]. Though the social norm of father involvement is changing, expectations and stereotypes depicting women as the main caregivers are communicated to children and therefore continue to persist [18]. For example, a recent pilot study conducted by the authors suggests that both men and women endorsed the belief that women are more knowledgeable, better suited, and naturally better than men at infant caregiving (*N* = 1474 undergraduate participants; 50% women). Men may internalize these stereotypes and prescriptions, leading to ambiguity surrounding their role during pregnancy. Because common gender prescriptions for men do not center around family life or childcare, men may perceive that there are lower, or unclear, expectations for their involvement during their partner’s pregnancy.

Broad societal expectations for men and women as parents allow men to be less involved, particularly during pregnancy. In addition, this norm may be reinforced through prenatal care settings that are not inclusive of men, because people avoid environments that signal a lack of “fit” [19]. Through structural elements (e.g., objects), environments can signal which social identities are valued and welcomed in a given space [20]. For example, science, technology, engineering and math educational settings that include stereotypical objects such as video games and Star Trek posters signaled to women that they were unwelcome compared to settings that included neutral objects such as water bottles [20]. Not feeling a sense of fit in an environment has important consequences, as people in such environments demonstrate lower trust, belonging, and motivation to participate [20]. Moreover, people seek out environments where they think they fit and avoid those that appear alienating [19]. For men, prenatal care spaces may not signal a fit to their social identities. Indeed, past work suggests that although many men would like to be involved in healthcare visits, they found the experiences to be focused only on women and lacking any content aimed towards men [21]. Qualitative research has found that men desire more educational materials aimed at their needs, and clarification of their role in prenatal care [22]. Indeed, gathering information about child care is an important aspect of prenatal preparation, and fathers who feel more prepared are more engaged with the child after birth [23]. Yet, in internet forums, men express feelings of being overlooked and ignored, suggesting that doctors have low or ambiguous expectations for their role during the pregnancy [24]. Current prenatal settings may improve the quality of care to expectant mothers by signaling to men that they are welcome and have an important supportive role to play in prenatal care.

Though some settings may be unwelcoming because they signal to specific people that their identity is devalued, this effect may be counteracted through the use of environmental safety cues. Safety cues can be structural elements added to a physical space that create a more welcoming environment for groups typically excluded [25]. For example, making a computer science environment less stereotypically male reduced the identity threat for women and increased their sense of belonging and interest in participating [20]. The same effect was found in virtual classrooms. Women who viewed virtual computer science classrooms that were designed to avoid computer science stereotypes reported greater interest and anticipated success in the course [26]. For men in prenatal care settings, structural cues may clarify their role and signal that they are welcome and play an important role [27]. Specifically, the environmental cues in a given space may signal what doctors expect of fathers in those settings. Therefore, we tested the hypothesis that including environmental safety cues for men in prenatal settings would clarify their role during the pregnancy by increasing the expectations that they believe doctors would have of fathers. Further, we expected that greater perceived expectations would be associated with greater comfort in the setting, greater confidence in their ability to be a good father, and greater reported intentions to be involved during the pregnancy.

### The present study

The present work tests whether creating a father-friendly prenatal care setting influences men’s perceptions of the environment and staff, including the perception of doctor’s beliefs about the role of fathers, feelings of comfort and fit in the environment, confidence, and behavioral intentions of prenatal involvement. Though prenatal involvement has been described in a variety of ways in the extant literature, the present studies focus on behavioral intentions to participate in the prenatal period by learning about infant care and engaging in positive health behaviors. This focus is consistent with definitions of father involvement as engaging in activities that lead to positive birth outcomes [1]. Including structural elements targeted to men in prenatal care settings communicates that men are welcome and expected during these medical visits, and may influence men to participate during the pregnancy. Below, we will report the results of a pilot field study exploring the current state of environmental cues in a sample of Obstetrics and Gynecology (OB/GYN) offices, as well as three randomized control studies examining the effect of father-friendly safety cues among an online sample of men (Study 1), a student sample in a laboratory setting (Study 2), and an online sample of current expectant fathers (Study 3). The research reported here was approved by Rutgers University’s Institutional Review Board, protocol # E15-756. Written consent was obtained from all participants. All measures, conditions, and data exclusions are reported here. The present studies were not pre-registered, but all data and materials may be found at: https://osf.io/n9dws

## Pilot study

### Method

#### Participants and procedure

A convenience sample of OB/GYN offices were selected in the northeast United States. Trained research assistants (*N* = 8; observing RAs) visited 21 different OB/GYN offices, including both private practices (76%) and hospital offices (24%). During their visit, observing RAs coded the environments based on a pre-determined set of questions wherein they reported on the décor in the waiting lobby and who was present at the time of their visit. These questions were created by the authors for the purpose of the present study based on past work examining the effect of environmental cues [20]. When permitted and appropriate, the observing RAs took photos of the lobby.

### Measures

#### Environmental cues

Observing RAs coded whether the office included objects, items, or décor aimed at men (3 items; magazines aimed at men, pictures of men alone or with babies, and information for fathers) and at women (3 items; magazines aimed at women, pictures of women alone or with babies, and information for mothers). These were summed for each location, creating a possible range of 0–3 where 3 indicates the most father-friendly (or mother-friendly) environment.

#### Identity safety

Observing RAs coded the father-friendliness (2 items; “How father-friendly is the waiting room?”, “How welcoming was the waiting room to fathers?”) of the waiting rooms using a scale of 1 (*not at all friendly/welcoming*) to 7 (*very friendly/welcoming*). We examined the reliability of these items through a correlation because there were less than three items, so a Cronbach’s alpha was not appropriate. Because the items were highly correlated (*r*(17) = .86, *p* < .001), we created a mean score of father-friendliness of the waiting rooms. RAs also coded the mother-friendliness (2 items; “How mother-friendly is the waiting room?”, “How welcoming was the waiting room to mothers?”) using the same scale. These items were also highly correlated (*r*(18) = .71, *p* = .001), and we created a mean score of mother-friendliness of the waiting rooms. Observing RAs also completed a one-item measure rating the femininity of the waiting room from 1 (*very masculine*) to 7 (*very feminine*). Lastly, observing RAs completed two binary items reporting whether there were men and women present in the waiting room.

## Results

### Analysis plan

Coders rated both mother-friendly and father-friendly cues in each office and rated the mother-friendliness and father-friendliness of each office, thus requiring a within-subjects analysis. Therefore, to test whether there were differences in the presence of father-friendly and mother-friendly environmental cues in the observed offices, we conducted a paired samples *t*-test. Similarly, we conducted a paired samples *t*-test to test for differences in how father-friendly and mother-friendly the observed offices were rated. We conducted a one sample *t*-test to test whether the décor was rated as more feminine than masculine because this was a single item. Lastly, we conducted a chi-squared test to test differences in the frequency of men and women seen in the offices.

## Results and discussion

The offices visited included more environmental cues for women (Mean [*M*] = 1.95, Standard Deviation [*SD*] = 1.12) than for men (*M* = 0.38, *SD* = 0.59), *t*(20) = 5.43, *p* < .001. Moreover, the offices were rated as more mother-friendly (*M* = 5.36, *SD* = 1.10) than father-friendly (*M* = 3.38, *SD* = 1.37), *t*(20) = 5.28, *p* < .001. However, the décor was rated close to average femininity and did not differ from the midpoint of the scale, *M* = 4.24, *SD* = 1.34, *t*(20) = 0.82, *p* = .424. Lastly, women were seen in the offices more often (women were seen in 14 offices, 67%) than men (men were seen in 4 offices; 19%), χ^2^ (*N* = 21, 1) = 9.64, *p* = .001. The results from this exploratory field study suggest that OB/GYN offices are typically more mother-friendly and consist of more environmental cues (magazines, pictures, etc.) that emphasize women. This confirms that the treatment-as-usual in OB/GYN offices are understandably focused on women, their primary patients, but this may come at the cost of fathers’ exclusion [27]. Given the results of the pilot study, Study 1 used an online paradigm where men viewed either a mother-friendly OB/GYN office (i.e., treatment-as-usual control) or an office that included a balance of mother- and father-friendly cues (hereafter, father-friendly office) and reported their impressions and future intentions.

## Study 1

### Method

#### Participants

We used a small-to-medium effect size to conduct a power analysis and determined that we needed 300 participants to achieve 80% power. Amazon Mechanical Turk workers (*N* = 301) completed a study about evaluating medical spaces and received a small token to compensate for their time. Amazon Mechanical Turk participants are reliable and attentive, and the participant pool is often more diverse than student samples [28–29]. Participants were excluded if they completed the survey multiple times (*n* = 2), answered the manipulation check question incorrectly on the second try (*n* = 1; see below), or marked their gender as female (*n* = 30). The final sample included 268 men. The mean age was 37.44 years, *SD*_*age*_ = 11.94 and 77% of participants were White, 9% Black, 7% Asian, 4% biracial, 1.5% Latino, 1.5% Native American or Alaskan Native, and < 1% selected other. The majority (*n* = 260, 97%) of participants were heterosexual. Over half of the sample (*n* = 151, 56%) had or were expecting children, and all of those who did not already have children indicated yes to the following question: “Do you want to have a child at some point in your life?”

### Procedure

Participants completed the study online. Using Qualtrics’ randomization setting, participants were randomly assigned to either view a video of a purported medical office that included mother cues only (control condition; *n* = 136) or both mother and father cues (intervention condition hereafter referred to as the father-friendly condition; *n* = 132). The videos lasted approximately a minute and a half, and showed the waiting room, the hallway, and the exam room. In the control condition, the video included pictures of mothers and infants as part of the décor in the waiting and patient areas as well as magazines aimed at women such as *Women’s Health*. In the father-friendly condition, the video included both father and mother friendly décor, including pictures of fathers with infants and magazines aimed at men such as *Men’s Health*. The video focused on the decorations and no people were shown in the videos. After viewing the video, participants answered one key manipulation check question (“What is one magazine that was included in the video?”). In the father-friendly condition the magazines aimed at women were not included as response options. Therefore, in each condition there was only one correct response, despite some overlap in the magazines across conditions. Participants who answered this question incorrectly (*n* = 68; 25%) were redirected to the video and instructed to watch it again. After re-watching the video, participants were asked the manipulation check question again. Next, participants completed the measures below in a randomized order. The measures were randomized in order to prevent an order effect emerging where participants’ responses on one measure systematically influence their responses on another. Lastly, participants were debriefed and compensated.

### Measures

#### Doctor expectations

Participants completed a 13-item measure to assess the expectations they perceived doctors at the office in the video would have of fathers. An example item is, “The doctors at this office think that fathers play an essential role in infant health during pregnancy.” Participants responded to items on a scale of 1 (*strongly disagree*/*not at all/do not expect this at all*) to 7 (*strongly agree/extremely/ highly expected*). All items were averaged into a reliable scale (α = .97).

#### Comfort

Participants reported how comfortable they would feel if they were expecting a child and attended the medical office in the video. They completed eight items such as “If I were expecting a child, I would feel comfortable going to this office with my partner” on a scale of 1 (*strongly disagree/ not at all*) to 7 (*strongly agree/extremely*). The items were averaged into a reliable scale (α = .92).

#### Parenting confidence

Participants’ confidence in their own ability to be parents was measured through six items such as, “I am confident in my ability to be a father.” Participants responded on a scale of 1 (*strongly disagree*) to 7 (*strongly agree*), and the items were averaged into a reliable scale (α = .93).

#### Learning intentions

Ten items measured participants’ intentions to learn more about caring for an infant if they were expecting a child with their partner. In other words, participants imagined a future pregnancy, and responded to items such as, “While my partner is pregnant, I will read the pregnancy books,” and “I would plan to be involved in the prenatal care.” Participants responded on a 7-point scale of 1 (*strongly disagree*) to 7 (*strongly agree*), and the items were averaged into a reliable scale (α = .93).

#### Consonant health behavior intentions

Participants responded to six items that measured their intentions to engage in supportive health behaviors with their partner during a future pregnancy. They responded to items such as, “During the pregnancy, I will not consume alcohol or tobacco products around my partner” on a scale of 1 (*strongly disagree*) to 7 (*strongly agree*). These items were averaged into a reliable scale (α = .83).

## Results

### Analysis plan

Studies 1–3 follow the same analytic plan. The preliminary analyses included two-way ANOVAs where parent status and condition were included as two independent variables. A separate two-way ANOVA was conducted for each dependent variable (doctor expectations, comfort, parenting confidence, learning intentions, and consonant health behavior intentions). This analysis tested potential differences between participants who do not have children and those who do or are currently expecting (i.e., parent status), allowing us to rule out interactions between parent status and condition. Next, we conducted independent samples *t*-tests to test for differences between men in the father-friendly versus treatment-as-usual control conditions for each measure. Lastly, we used the PROCESS macro for SPSS to test whether doctor expectations mediated the effect of condition on each outcome [30]. We examined the bootstrapped estimate of 10,000 resamples of the indirect effect to determine whether the indirect effect was significant.

### Preliminary results

There was no significant interaction between condition and parent status for doctor expectations, *F*(1, 264) = .20, *p* = .654, comfort, *F*(1, 264) = .19, *p* = .666, confidence, *F*(1, 264) = 1.29, *p* = .257, learning intentions, *F*(1, 264) = 1.42, *p* = .234, or consonant health behavior intentions, *F*(1, 264) = 1.29, *p* = .257. This suggests that the effects of condition did not vary by participants’ experience with parenting and thus, this factor was not included in subsequent analyses.

### Condition effects

There was a significant effect of condition on doctor’s expectations such that men in the father-friendly condition believed that doctors would hold fathers to higher expectations (*M* = 5.92, *SD* = 0.99) than the control condition (*M* = 4.95, *SD* = 1.41), *t*(266) = 6.51, *p* < .001, *d* = 0.80, 95% Confidence Interval (CI) = [0.55, 1.04]. Similarly, men reported they would feel more comfortable in the father-friendly office condition (*M* = 5.87, *SD* = 0.98) than in the control condition (*M* = 5.26, *SD* = 1.18), *t*(266) = 4.65, *p* < .001, *d* = 0.56, 95% CI = [0.32, 0.81]. There were no direct effects of condition on parenting confidence, learning intentions, or consonant health behaviors, *t*s < 1.14, *p*s > .255.

### Mediation analyses

**Fig 1:**
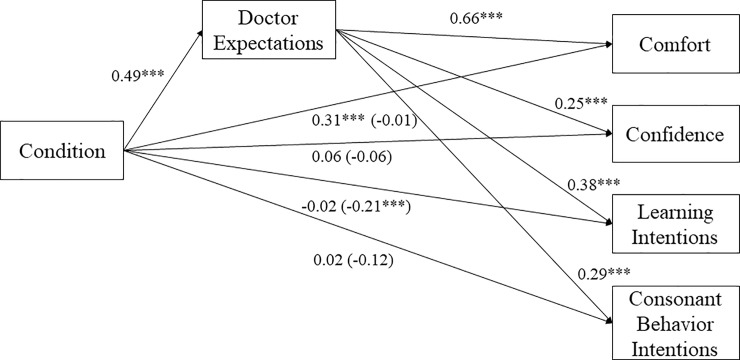
The indirect effect of condition on each outcome through doctor expectations in Study 1. *Note*. We tested each mediation outcome separately, but we present the results together for ease of interpretation.

We tested whether doctor expectations mediated the effect of condition (1 = *father-friendly*; -1 = *control*) on comfort, confidence, and intentions (see Fig 1). We first examined the indirect effect of condition on comfort through doctor expectations. The indirect effect was significant, *B* = 0.32, *SE* = 0.05, 95% CI = [0.23, 0.43], suggesting men in the father-friendly condition believed doctors would have higher expectations of fathers, which predicted greater comfort in that medical office. Similarly, the indirect effect of condition on confidence, *B* = 0.12, *SE* = 0.03, 95% CI = [0.06, 0.19] was significant through doctor expectations. Lastly, the indirect effect was significant on learning intentions, *B* = 0.18, *SE* = 0.03, 95% CI = [0.13, 0.26], and consonant behavioral intentions, *B* = 0.14, *SE* = 0.03, 95% CI = [0.09, 0.21]. Men in the father-friendly condition reported greater perceived doctor expectations, which predicted greater confidence in their abilities to be good fathers, greater intentions to learn more about pregnancy and children in anticipation of a child, and greater intentions to encourage their partner through consonant healthy behaviors. All results reported did not change if we excluded gay or bisexual participants.

The results of Study 1 suggest that men were responsive to environmental cues that raised expectations of fathers’ involvement. Men also reported having a higher level of comfort than the control condition. The mediation models suggest that men would respond positively to these higher expectations, as those in the father-friendly condition reported that they would feel more comfortable, confident, and would engage in greater learning and consonant health behaviors. Although we took care to ensure that the online experience was as realistic as possible for participants (i.e., watching a video rather than viewing static images, and including manipulation check questions), it is more engaging to be immersed in these environments firsthand. Therefore, Study 2 sought to replicate and expand Study 1 by creating a physical medical office space that participants could experience themselves.

## Study 2

### Method

#### Participants

We recruited a convenience sample of 250 male participants from the psychology subject pool at Rutgers University to participate in a study on medical environments. We excluded participants who failed both manipulation check questions (*n* = 22; see below), leaving a final sample size of 228 men. A sensitivity power analysis suggests that this sample size provided 80% power to detect effects with a minimum effect size of *d* = 0.37. The average age was 19.31 (*SD* = 2.94) and the sample was 48% Asian, 27% White, 11% multiracial, 7% Latino, 3% Black, 2% Middle Eastern, and 2% selected other. Only 2 participants (1% of sample) had children, 96% wanted or maybe wanted to have children in the future, and 91% were heterosexual.

### Procedure and measures

The study was held in a research environment on a university campus that mirrors a doctor’s office setting with a waiting room and patient clinical space. Importantly, it was the same environment from the videos in Study 1. Participants were brought into a small lobby, which was set up to be either father-friendly (*n* = 120) or the treatment-as-usual control (*n* = 108). As in Study 1, the father-friendly condition included objects such as pictures of both men with children and women with children, as well as magazines geared towards men, and an informational brochure for fathers. The control condition did not include pictures of men, and included only magazines geared towards women, and an informational brochure for mothers. Participants were shown around the space and were instructed to imagine themselves waiting for an appointment at the office of an obstetrician-gynecologist. This was defined as a doctor that specializes in reproductive health, especially during pregnancies and birth. After three minutes, participants remained in the environment and completed two manipulation check questions (“What is one magazine that you saw in the office waiting room?” and “What color is the pamphlet on the table?”). In this study, they did not have the opportunity to correct their answers. Lastly, participants competed the same measures of doctor expectations (α = .97), comfort (α = .91), parenting confidence (α = .83), learning intentions (α = .87), and consonant behaviors (α = .66) from Study 1.

## Results

Preliminary analyses testing for differences by parenting status were not conducted because there were only 2 participants who already had children. We conducted independent *t*-tests to examine the effect of condition on each variable. There was an effect of condition on doctor’s expectations such that men in the father-friendly condition reported higher doctor expectations of fathers (*M* = 6.13, *SD* = 0.72) compared to control (*M* = 4.46, *SD* = 1.66), *t*(226) = 10.02, *p* < .001, *d* = 1.33, 95% CI = [1.04, 1.62]. Similarly, men in the father-friendly waiting room reported they would feel more comfortable (*M* = 6.10, *SD* = 0.74) in that space than men in the control condition (*M* = 5.14, *SD* = 1.17), *t*(226) = 7.50, *p* < .001, *d* = 0.99, 95% CI = [0.72, 1.27]. There was also an effect on learning intentions. Men in the father-friendly condition reported higher learning intentions (*M* = 6.41, *SD* = 0.56) than men in the control condition (*M* = 6.16, *SD* = 0.82), *t*(226) = 2.69, *p* = .008, *d* = 0.36, 95% CI = [0.09, 0.62]. There were no effects of condition on parenting confidence or consonant health behaviors, *t*s < 1.23, *p*s > .220.

### Mediation analyses

**Fig 2:**
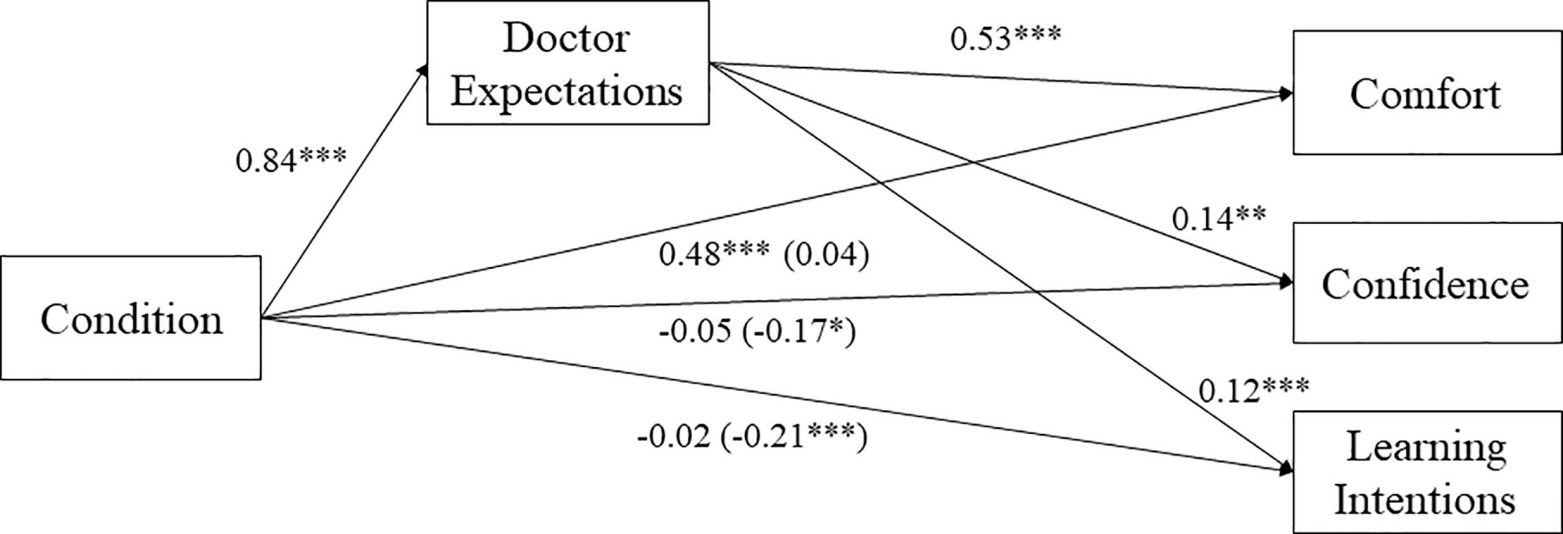
The indirect effect of condition on each outcome through doctor expectations in Study 2. *Note*. We tested each mediation outcome separately, but we present the results together for ease of interpretation.

Similar to Study 1, we tested whether doctor expectations mediated the effect of condition (1 = *father-friendly*; -1 = *control*) on comfort, parenting confidence, learning intentions and consonant behavioral intentions (see Fig 2). Using the PROCESS macro for SPSS, we examined the bootstrapped estimate of 10,000 resamples of the indirect effect of condition on each outcome through doctor expectations [30]. We first examined the indirect effect of condition on comfort. The indirect effect was significant, *B* = 0.44, *SE* = 0.05, 95% CI = [0.34, 0.55], suggesting that in the father-friendly condition men believed doctors would have higher expectations of fathers, which predicted greater comfort in that medical office. Similarly, the indirect effects of condition on confidence, *B* = 0.11, *SE* = 0.04, 95% CI = [0.04, 0.20], and learning intentions, *B* = 0.10, *SE* = 0.03, 95% CI = [0.05, 0.17], were significant through doctor expectations. However, the indirect effect of condition on consonant behavioral intentions was not significant, *B* = -0.002, *SE* = 0.03, 95% CI = [-0.06, 0.05]. All results reported here did not change if we excluded gay or bisexual participants and those who did not or were not sure they wanted to have children in the future.

Study 2 replicated and extended Study 1 by adding more ecological validity. Study 2 also tested the same hypotheses among a different sample of participants. The college student sample was younger than the online sample and included significantly fewer participants who already had experience in prenatal settings. The results provide converging evidence that father-friendly environments are believed to be more comfortable for men and shift their perceptions of doctors’ expectations of father’s involvement. Moreover, expectations appear to explain why father-friendly environments comfort and have the potential to motivate men to be more involved fathers in the future.

Although Studies 1 and 2 support our main hypotheses in both virtual and real settings, we wanted to test this effect among our population of interest: expectant fathers. Study 3 used the same online paradigm from Study 1 but only included men who were expecting a child at the time of the study. We anticipated replicating the findings of Study 1 given that these men were expected to be especially sensitive to these cues.

## Study 3

### Method

#### Participants

Given the effect sizes of the previous studies (average effect *d* = 0.62), we aimed to recruit at least 100 expectant men for a study on “Medical Spaces.” Men were recruited using an online panel service. The original sample included 141 men, but we excluded 30 men for non-sense responses to open-ended questions, which signaled that they were not completing the survey honestly. This left a final sample of 111 men. A sensitivity power analysis suggests that this sample size provided 80% power to detect effects with a minimum effect size of *d* = 0.54. The average age was 31.55 (*SD* = 8.20), the sample was 78% white, 8% Asian, 5% Black, 4.5% Latino, and 4.5% Multiracial, 96% heterosexual, and 27% did not already have any children.

### Procedure and measures

Study 3 used the same procedure as Study 1, and participants completed the study online. Participants viewed the same videos (*n* = 61 father-friendly; *n* = 50 treatment-as-usual) and completed the same manipulation check question. Those who missed the manipulation check question the first time (*n* = 17) were able to re-watch the video and answer again. All participants answered the question correctly on the second try. Participants next completed the same measures of doctor-expectations (α = .97), comfort (α = .95), confidence (α = .91), learning intentions (α = .91), and consonant health behavioral intentions (α = .76) from Study 2. Additionally, participants in this study reported whether they had accompanied the mother of their child to an OB/GYN appointment. Those who had (*N* = 105, 95% of sample) completed five items reporting how comfortable they felt in that situation (e.g., “I felt comfortable going to this office with my partner;” α = .92). Participants also indicated whether the décor at the office they visited included any of six objects. The responses to three key items (magazines aimed at men, pictures of men alone or with babies, and information for fathers about parenting, pregnancy, childbirth, etc.) were summed for each participant, creating a possible range of 0–3 where 3 indicates the most father-friendly environment. Lastly, participants responded to six items indicating how friendly the staff was to them at the appointment (e.g., “How much did you feel included in the conversation with the medical staff at this office?”; α = .80). Participants reported high levels of comfort during the OB/GYN visit, *M* = 6.14, *SD* = 0.88. Lastly, participants were debriefed and compensated.

## Results

Preliminary analyses testing for differences by parenting status were not conducted because all participants were currently expecting a child and therefore shared the same parenting status. We conducted independent *t*-tests to examine the effect of condition on each variable. Expectant fathers who viewed the father-friendly medical office reported higher doctor expectations (*M* = 6.06, *SD* = 0.86) compared to control (*M* = 5.03, *SD* = 1.68), *t*(109) = 4.15, *p* < .001, *d* = 0.79, 95% CI = [0.40, 1.18]. Men in the father-friendly condition also reported they would feel more comfortable (*M* = 6.03, *SD* = 0.87) in that space than men in the control condition (*M* = 5.33, *SD* = 1.53), *t*(109) = 3.03, *p* = .003, *d* = 0.58, 95% CI = [0.20, 0.96]. There were no effects of condition on parenting confidence, learning intentions, or consonant health behaviors, *t*s < 0.42, *p*s > .679.

### Mediation analyses

**Fig 3:**
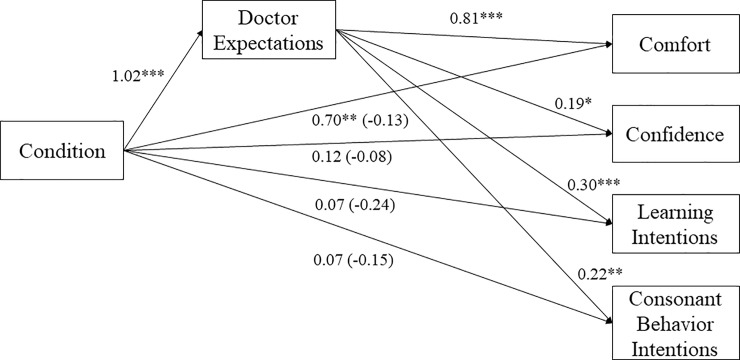
The indirect effect of condition on each outcome through doctor expectations in Study 3. *Note*. We tested each mediation outcome separately, but we present the results together for ease of interpretation.

As in the previous studies, we tested whether doctor expectations mediated the association between condition (1 = *father-friendly*; -1 = *control*) and each outcome (see Fig 3). The indirect effect of condition on comfort through doctor expectations was significant, *B* = 0.83, *SE* = 0.22, 95% CI = [0.44, 1.29], suggesting in the father-friendly condition men believed doctors would have higher expectations of fathers, which predicted greater comfort in that medical office. The indirect effects of condition on confidence, *B* = 0.20, *SE* = 0.07, 95% CI = [0.08, 0.39], learning intentions, *B* = 0.31, *SE* = 0.10, 95% CI = [0.15, 0.57], and consonant behavioral intentions, *B* = 0.23, *SE* = 0.09, 95% CI = [0.08, 0.44] were significant. Men in the father-friendly condition reported greater doctor expectations, which predicted greater confidence, learning intentions and consonant health behavior intentions. All results reported did not change if we excluded gay or bisexual participants.

## Discussion

The present studies tested an affordable and scalable intervention to increase men’s comfort and perceived expectations of involvement in prenatal care settings. Using both online and in-person methodologies, the findings of three studies suggest that including environmental cues that signal inclusion to men can alter the expectations that men believe doctors have of their involvement. In turn, this new norm is then associated with greater comfort in the doctor’s office, more confidence in their ability to be a good parent, and greater reported intentions to learn about pregnancy and engage in healthy behaviors along with their partner. Study 2 demonstrated this effect in an immersive environment, and Study 3 provided a final replication among a sample of the population of interest: expectant fathers. Overall, including environmental cues like men’s magazines and pictures of fathers in the décor led participants to think that doctors in that office would expect men to be more involved in the prenatal period. Participants responded to this altered social norm by reporting greater intentions to be involved.

Across the studies, there were no direct effects of condition on parenting confidence, learning intentions or consonant health behaviors. This suggests that viewing a father-friendly medical space did not directly influence men’s behavioral intentions or confidence. This may be expected, given that the cues included in the office were not promoting specific behaviors. Rather, the space signaled to men what doctors may expect of them, which then predicted greater confidence and behavioral intentions. These findings are consistent with past work, which suggests that environmental cues can increase the ambient belonging of group members traditionally excluded from those spaces [20]. However, the present studies extend existing work by examining a novel mediator of this association. The present studies found support that environmental cues influence behavioral intentions, confidence, and comfort through increased perceived doctors’ expectations, while the existing literature has largely focused on sense of belonging and identification as mediators [20,31]. These findings suggest that the environmental cues helped men deduce what role is expected of them in that setting, leading to greater feelings of inclusion and motivation to be involved.

The findings were consistent across the three studies with two minor exceptions. In Study 2, there was no significant mediation effect for consonant health behaviors, and there was a significant direct effect on learning intentions. The sample and methodology differed in this study because the participants were undergraduate students and they visited the office in person rather than viewing a video online. These variations may account for slightly different results. However, given the replication of the results for the remaining variables across three studies, we urge caution in interpreting these differences.

These findings are consistent with social psychological theory and research suggesting that environmental cues can signal inclusion based on social identities [19–20]. Given that fathers have reported a desire to clarify their role in prenatal care and receive more educational materials [22], the present studies present a feasible intervention to help fathers feel that they are included and expected to participate in prenatal care. Altering the perceived expectations helps create a new social norm wherein fathers are involved in prenatal care and that can motivate fathers to behave in line with these expectations. Involvement including engaging in supportive health behaviors, such as abstaining from alcohol and tobacco use is especially important as fathers’ health behaviors can strongly influence women’s abstinence during pregnancy [32–33]. Thus, the present results have benefits for both fathers and mothers.

### Implications and applications

The findings suggest that OB/GYN health care environments may be easily improved by including cues that signal men are welcome and invited. Though situational cues do not necessarily mean that men will be actively included by the doctors in the visit, and the desire for men’s inclusion and participation also varies by woman [27], the results of the present studies suggest the cues may encourage men to attend prenatal visits. Currently, many men report that their role during the pregnancy is unclear [22]. The existing medical model infrequently includes information aimed towards men during prenatal visits. For example, a recent systematic review found only 19 perinatal parent education programs that involved men [34]. The lack of education and content aimed towards men may leave many fathers with unanswered questions and feeling excluded from the process [27]. This is ultimately a disservice to both parents and the child, as work suggests involving parents in the prenatal period engenders many positive benefits [34]. Moreover, men who become involved during the pregnancy are more likely to continue to be involved later in the child’s life, bringing forth continued benefits [4]. Thus, the present results provide an easy and affordable intervention that may bring about important and long-lasting changes.

### Limitations and future directions

The studies are limited in that behavioral intentions were measured rather than actual behaviors. Though evidence supports that behavioral intentions are reliably related to behavior [35], future work should examine actual behavioral outcomes to better gauge the possible impact of such an intervention. Moreover, given that the primary population of interest was men, the present studies did not include women. However, future studies may also explore women’s perceptions of prenatal care offices that include fathering cues. Past work suggests majority group members may feel excluded or believe it is unfair when environments are changed to include minority group members [36]. However, given that both spaces included cues to welcome mothers, we do not expect women to feel excluded. Yet, women may vary in the extent to which they want their partner to be involved in prenatal care, an individual difference that may moderate women’s responses to a father-friendly office. Lastly, the present studies included the same father-friendly cues. However, future studies may examine the effects of more direct cues such as specific content guided towards men. It is possible that a more direct message aimed towards men may engender similar increased behavior intentions, but possibly through a different mechanism. Men in those settings may feel more efficacious or knowledgeable, which could influence their intentions to be involved.

### Conclusions

The present studies demonstrated that including environmental cues that feature men in prenatal care settings may influence men’s beliefs about doctors’ expectations. In father-friendly settings, men believed that doctors would expect them to be more involved during the pregnancy, and in turn reported greater comfort, confidence, and intentions to learn about pregnancy and engage in healthy prenatal behaviors. Given the persistent benefits of early father involvement, and the current lack of clear roles communicated to men in the prenatal period, the results highlight an important and feasible way to increase men’s prenatal involvement.

## References

[1] Alio AP, Bond MJ, Padilla YC, Heidelbaugh JJ, Lu M, Parker WJ. Addressing policy barriers to paternal involvement during pregnancy. Matern Child Health J. 2011;15: 425–430. 10.1007/s10995-011-0781-1 21472512

[2] Alio AP, Salihu HM, Kornosky JL, Richman AM, Marty PJ. Feto-infant health and survival: Does paternal involvement matter?. Matern Child Health J. 2010;14: 931–937. 10.1007/s10995-009-0531-9 19826935

[3] Lee SJ, Sanchez DT, Grogan-Kaylor A, Lee JY, Albuja A. Father early engagement behaviors and infant low birth weight. Matern Child Health J. 2018;22: 1407–1417. 10.1007/s10995-018-2521-2 29564605

[4] Cabrera NJ, Fagan J, Farrie D. Explaining the long reach of fathers’ prenatal involvement on later paternal engagement. J Marriage Fam. 2008;70: 1094–1107. 10.1111/j.1741-3737.2008.00551.x 20165557 PMC2822357

[5] Cook JL, Jones RM, Dick AJ, Singh A. Revisiting men’s role in father involvement: The importance of personal expectations. Fathering. 2005;3: 165–178.

[6] Martin LT, McNamara MJ, Milot AS, Halle T, Hair EC. The effects of father involvement during pregnancy on receipt of prenatal care and maternal smoking. Matern Child Health J. 2007;11: 595–602. 10.1007/s10995-007-0209-0 17557201

[7] Johnson WE. Paternal involvement among unwed fathers. Child Youth Serv Rev. 2001;23: 513–536.

[8] Cheng ER, Rifas-Shiman SL, Perkins ME, Rich-Edwards JW, Gillman MW, Wright R, et al The influence of antenatal partner support on pregnancy outcomes. J Womens Health. 2016;25: 672–679. 10.1089/jwh.2015.5462 PMC4985003 26828630

[9] Fagan J, Barnett M, Bernd E, Whiteman V. Prenatal involvement of adolescent unmarried fathers. Fathering. 2003;1: 283–301.

[10] Coltrane S, Parke RD, Adams M. Complexity of father involvement in low‐income Mexican American families. Fam Relat. 2004;53: 179–189.

[11] Eagly AH, Wood W. The origins of sex differences in human behavior: Evolved dispositions versus social roles. Am Psychol. 1999;54: 408–423.

[12] Prentice DA, Carranza E. What women and men should be, shouldn't be, are allowed to be, and don't have to be: The contents of prescriptive gender stereotypes. Psychol Women Q. 2002;26: 269–281.

[13] Rudman LA, Moss-Racusin CA, Phelan JE, Nauts S. Status incongruity and backlash effects: Defending the gender hierarchy motivates prejudice against female leaders. J Exp Soc Psychol. 2012;48: 165–179.

[14] Park B, Smith JA, Correll J. The persistence of implicit behavioral associations for moms and dads. J Exp Soc Psychol. 2010;46: 809–815.

[15] Craig L. Does father care mean fathers share? A comparison of how mothers and fathers in intact families spend time with children. Gend Soc. 2006;20: 259–281.

[16] Wall G, Arnold S. How involved is involved fathering? An exploration of the contemporary culture of fatherhood. Gend Soc. 2007;21: 508–27.

[17] Lamb ME. The history of research on father involvement: An overview. Marriage Fam Rev. 2000;29: 23–42.

[18] Croft A, Schmader T, Block K, Baron AS. The second shift reflected in the second generation: Do parents’ gender roles at home predict children’s aspirations?. Psychol Sci. 2014;25: 1418–1428. 10.1177/0956797614533968 24890499

[19] Schmader T, Sedikides C. State authenticity as fit to environment: The implications of social identity for fit, authenticity, and self-segregation. Pers Soc Psychol Rev. 2017;22: 228–259. 10.1177/1088868317734080 28975851

[20] Cheryan S, Plaut VC, Davies PG, Steele CM. Ambient belonging: How stereotypical cues impact gender participation in computer science. J Pers Soc Psychol. 2009;97: 1045–1060. 10.1037/a0016239 19968418

[21] Deave T, Johnson D. The transition to parenthood: what does it mean for fathers?. J Adv Nurs. 2008;63: 626–633. 10.1111/j.1365-2648.2008.04748.x 18808584

[22] Steen M, Downe S, Bamford N, Edozien L. Not-patient and not-visitor: A metasynthesis fathers’ encounters with pregnancy, birth and maternity care. Midwifery. 2012;28: 422–31. 10.1016/j.midw.2011.06.009 21820778

[23] Plantin L, Olykoya A, Ny P. Positive health outcomes of fathers’ involvment in pregnancy and childbirth paternal support: A scope study literature review. Fathering. 2011;9: 87–102.

[24] Salzmann‐Erikson M, Eriksson H. Fathers sharing about early parental support in health‐care‐virtual discussions on an Internet forum. Health Soc Care Community. 2013;21: 381–390. 10.1111/hsc.12028 23496139

[25] Purdie-Vaughns V, Steele CM, Davies PG, Ditlmann R, Crosby JR. Social identity contingencies: How diversity cues signal threat or safety for African Americans in mainstream institutions. J Pers Soc Psychol. 2008 4;94: 615–630. 10.1037/0022-3514.94.4.615 18361675

[26] Cheryan S, Meltzoff AN, Kim S. Classrooms matter: The design of virtual classrooms influences gender disparities in computer science classes. Comput Educ. 2011;57: 1825–1835.

[27] Draper H, Ives J. Men’s involvement in antenatal care and labour: Rethinking a medical model. Midwifery. 2013;29: 723–729. 10.1016/j.midw.2013.02.007 23522667

[28] Hauser DJ, Schwarz N. Attentive Turkers: MTurk participants perform better on online attention checks than do subject pool participants. Behav Res Methods. 2016;48: 400–407. 10.3758/s13428-015-0578-z 25761395

[29] Rouse SV. A reliability analysis of Mechanical Turk data. Comput Human Behav. 2015;43: 304–307.

[30] Hayes AF. PROCESS: A versatile computational tool for observed variable mediation, moderation, and conditional process modeling. Available from http://www.afhayes.com/public/process2012.pdf

[31] Stout JG, Dasgupta N, Hunsinger M, McManus MA. STEMing the tide: using ingroup experts to inoculate women's self-concept in science, technology, engineering, and mathematics (STEM). J Pers Soc Psychol. 2011;100: 255–270. 10.1037/a0021385 21142376

[32] Albuja AF, Sanchez DT, Lee SJ, Lee JY. Early paternal support behaviors moderate consonant smoking among unmarried parents. J Stud Alcohol Drugs. 2019;80:129–133. 10.15288/jsad.2019.80.129 30807285 PMC6396514

[33] Appleton PL, Pharoah PO. Partner smoking behaviour change is associated with women's smoking reduction and cessation during pregnancy. Br J Health Psychol. 1998;3: 361–374.

[34] Lee JY, Knauer HA, Lee SJ, MacEachern MP, Garfield CF. Father-inclusive perinatal parent education programs: A systematic review. Pediatrics. 2018:e20180437 10.1542/peds.2018-0437 29903835

[35] Webb TL, Sheeran P. Does changing behavioral intentions engender behavior change? A meta-analysis of the experimental evidence. Psychol Bull. 2006;132: 249–268. 10.1037/0033-2909.132.2.249 16536643

[36] Dover TL, Major B, Kaiser CR. Members of high-status groups are threatened by pro-diversity organizational messages. J Exp Soc Psychol. 2016;62: 58–67.

